# Supplementary material for: Neonatal Plasma Exosomes Contribute to Endothelial Cell-Mediated Angiogenesis and Cardiac Repair after Acute Myocardial Infarction
Source: Int J Mol Sci. 2023 Feb 6;24(4):3196. doi: 10.3390/ijms24043196 (PMC9959818; doi:10.3390/ijms24043196)
Supplement: Supplementary file 1 [file ijms-24-03196-s001.zip › ijms-2180014-supplemental figures_proof.pdf]

Supplementary Figure S1

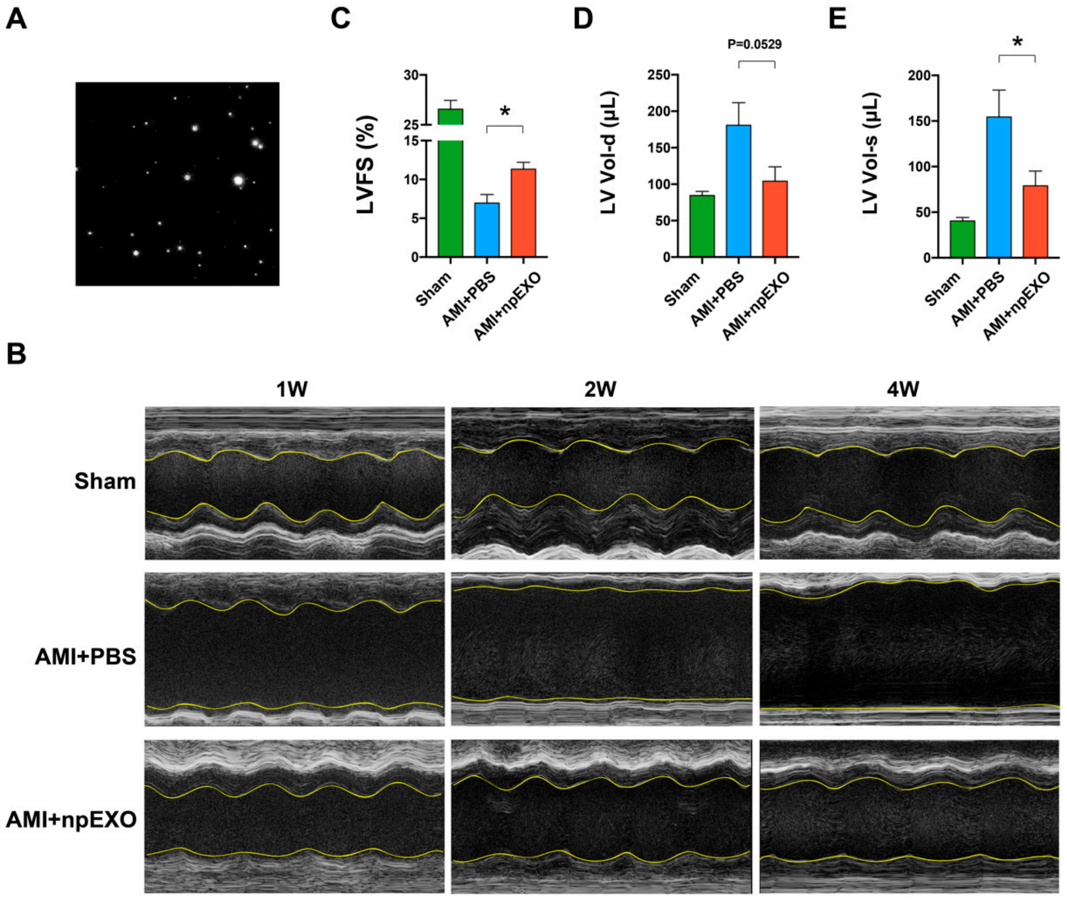

Supplementary Figure. S1 npEXO characteristics and cardiac function detection by echocardiography after npEXO treatment.

A Representative image of npEXO by NAT analysis.

B Representative echocardiography images at 1 week, 2 weeks, and 4 weeks after MI and npEXO treatment.

C-E Statistics of LVFS (C), LV Vol-d (D), and LV Vol-s (E) at 4 weeks (Sham, n=3; AMI+PBS, n=3; AMI+npEXO, n=3).

Supplementary Figure S2

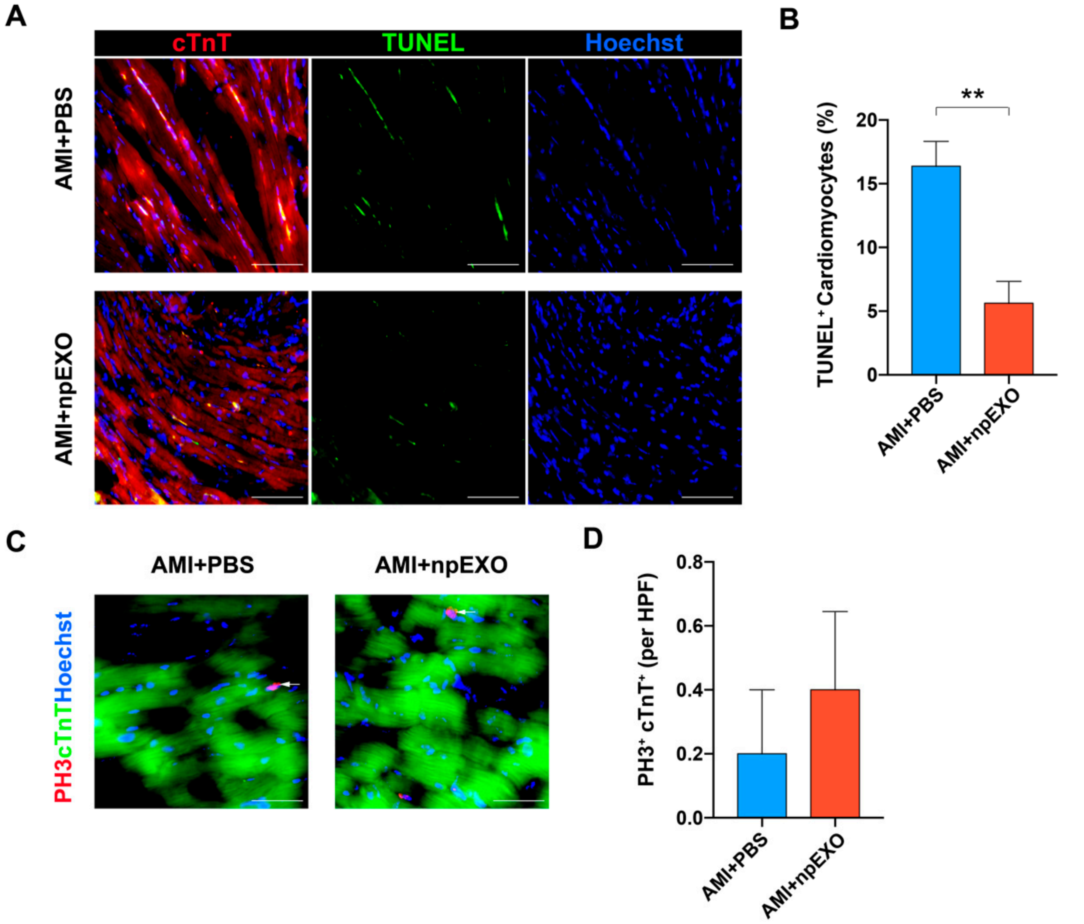

Supplementary Figure. S2 The CM apoptosis and proliferation after AMI and npEXO treatment.

A The CM apoptosis was detected by TUNEL staining 3 days after AMI (Scale bars, 200 μm).

B Statistics of TUNEL-positive CMs (AMI+PBS, n=3; AMI+npEXO, n=5).

C Representative image of PH3/cTnT double-positive CMs (white arrows, the PH3/cTnT double-positive CMs; Scale bars, 50 μm).

D Statistics of PH3/cTnT double-positive CMs, HPF: High power field, (AMI+PBS, n=5; AMI+npEXO, n=5).

Supplementary Figure S3

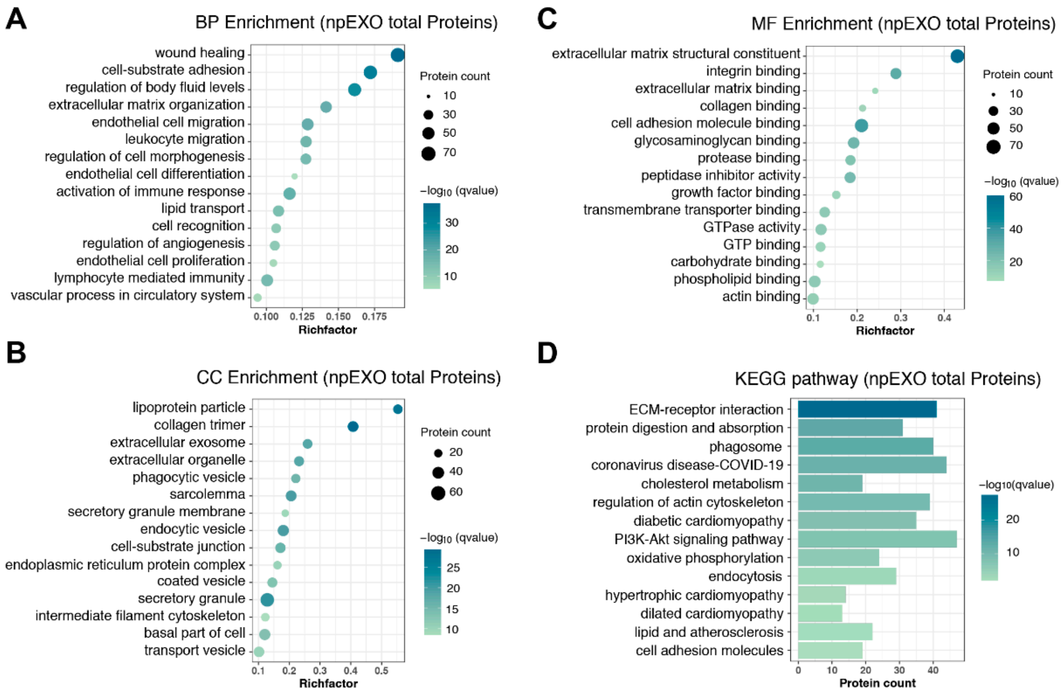

Supplementary Figure. S3 GO analyses of total npEXO proteins.

A GO analysis (BP enrichment) of npEXO proteins.

B GO analysis (CC enrichment) of npEXO proteins.

C GO analysis (MF enrichment) of npEXO proteins.

D KEGG pathway enrichment analysis of npEXO proteins.

Supplementary Figure S4

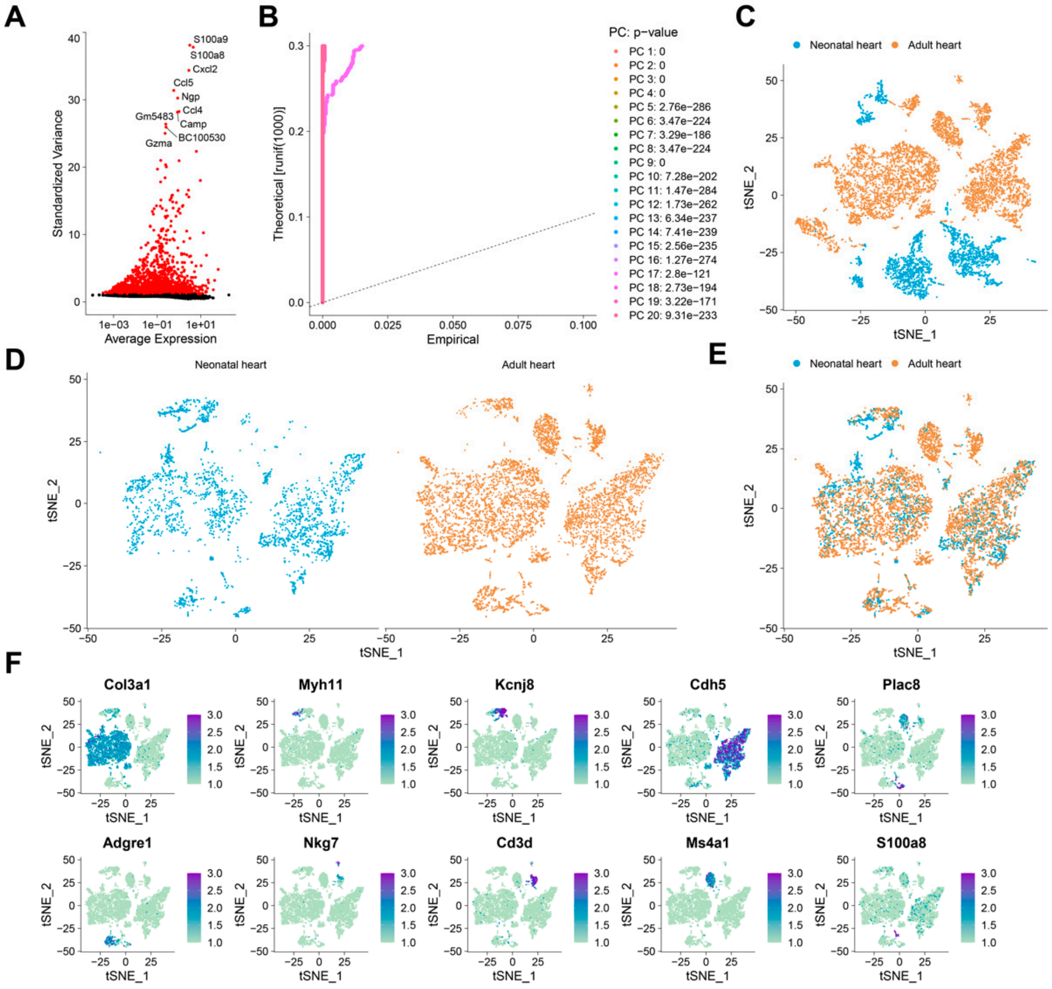

Supplementary Figure. S4 scRNA-Seq analysis revealed the major non-CM types in neonatal and adult hearts.

A-B Gene filtering (A) and PCA clustering (B) of the neonatal and adult heart non-CM gene expression matrix.

C tSNE plot of unintegrated non-CMs under different conditions shown in different colors.

D Separated tSNE plots of integrated non-CMs in the neonatal and adult heart.

E tSNE plot of integrated non-CMs under different conditions shown in different colors.

F tSNE plot showing the representative marker genes for all defined non-CM types.

Supplementary Figure S5

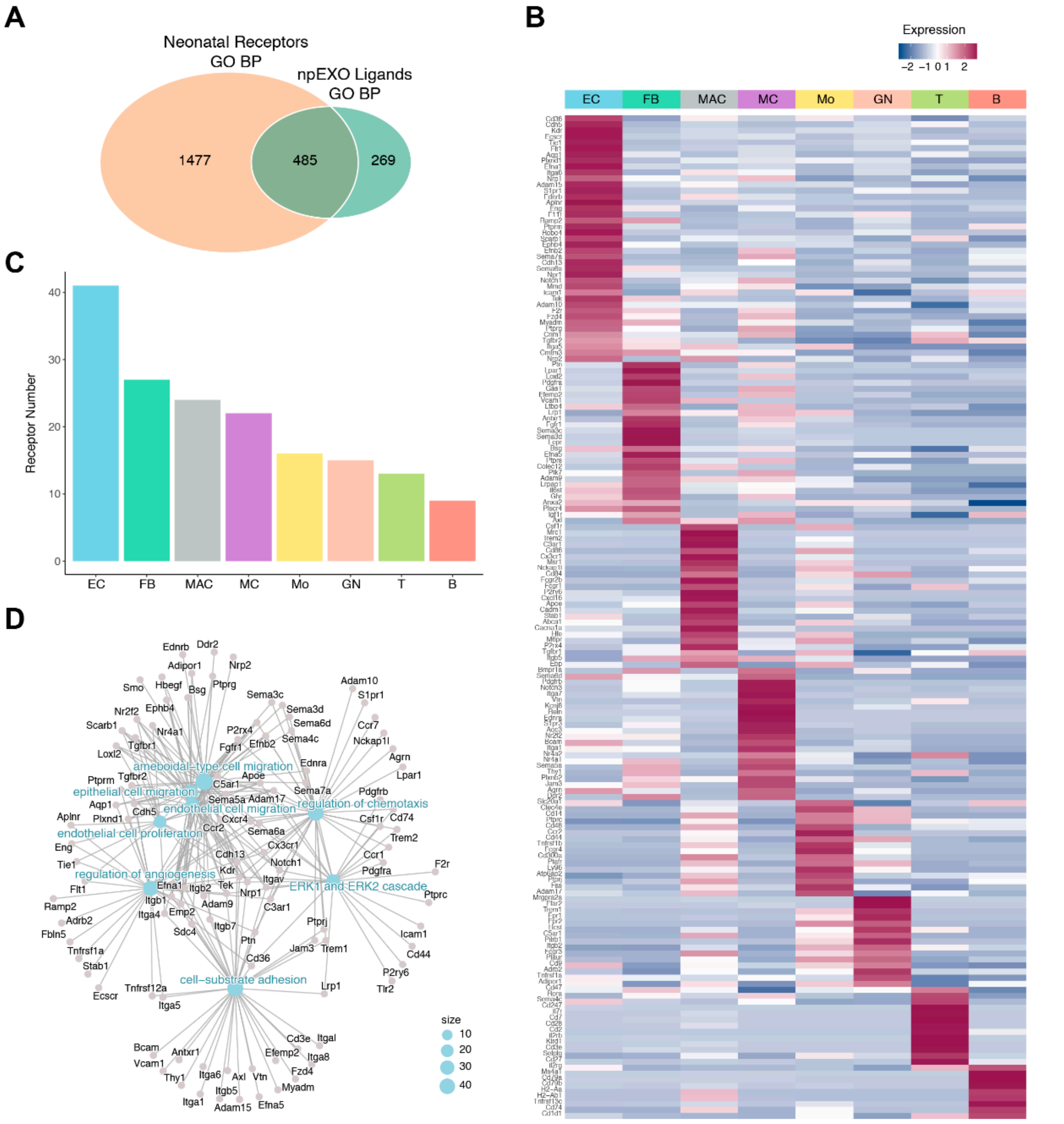

Supplementary Figure. S5 The ECs were the main non-CM cell type in neonatal mouse heart to receive npEXO ligands.

A Venn diagram showing GO results (BP enrichment) of npEXO ligand against those of non-CM receptors in the neonatal heart.

B Heatmap displaying the expression of receptor genes involved in npEXO and non-CM communication network of neonatal heart. Clusters are assembled by GO analysis. The significantly enriched items and representative genes are shown on the left.

C Sum of all communication network receptor genes expressed in each non-CM population.

D Correlation analysis showed pathways and receptors expressed in ECs in neonatal hearts.

Supplementary Figure S6

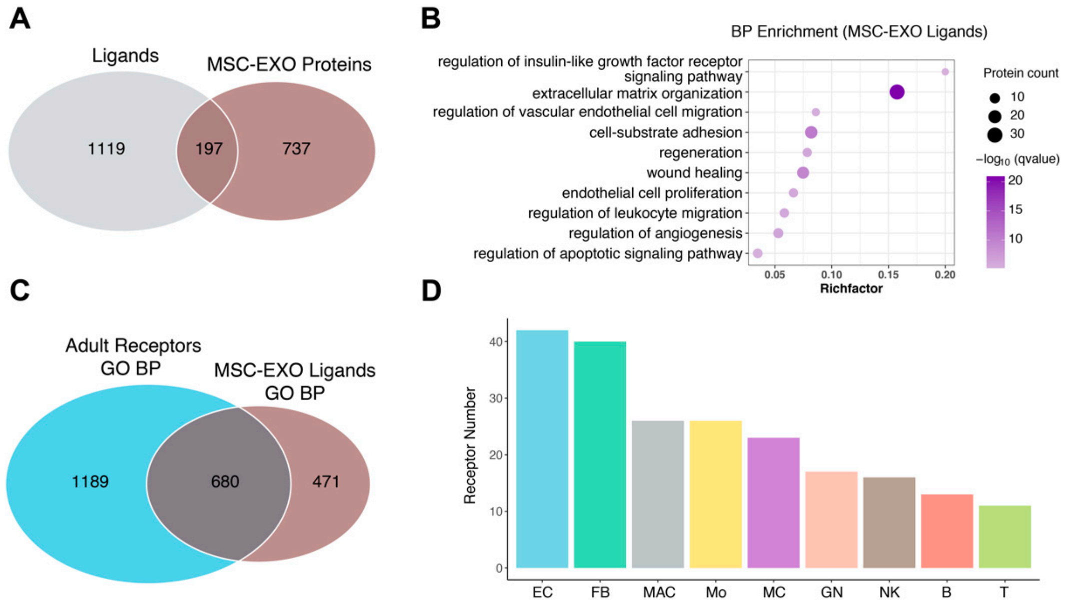

Supplementary Figure. S6 Construction of communication network between MSC-EXO and non-CMs in the adult heart.

A Venn diagram of MSC-EXO proteins against the ligand dataset.

B GO analysis (BP enrichment) of MSC-EXO ligand proteins.

C Venn diagram showing GO results (BP enrichment) of MSC-EXO ligands against those of non-CM receptors in adult hearts.

D Sum of all communication network receptor genes expressed in each non-CM population.

Supplementary Figure S7

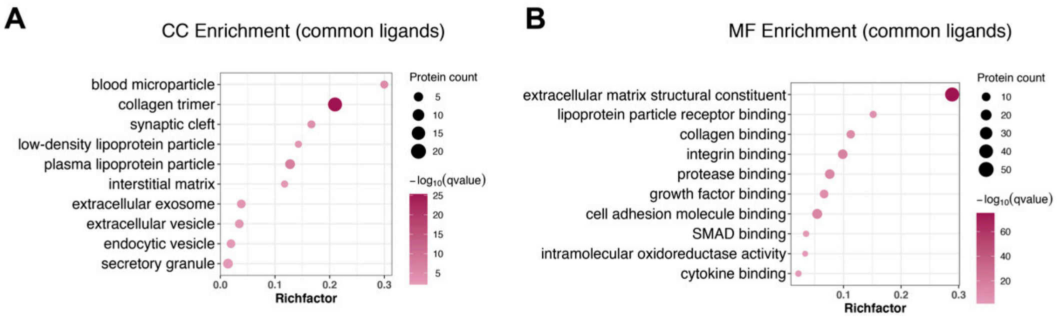

Supplementary Figure. S7 GO analyses of common ligands shared by npEXO and MSC-EXO.

A-B GO analysis (CC (A) and MF (B) enrichment) of common ligands shared by npEXO and MSC-EXO.

Supplementary Figure S8

A

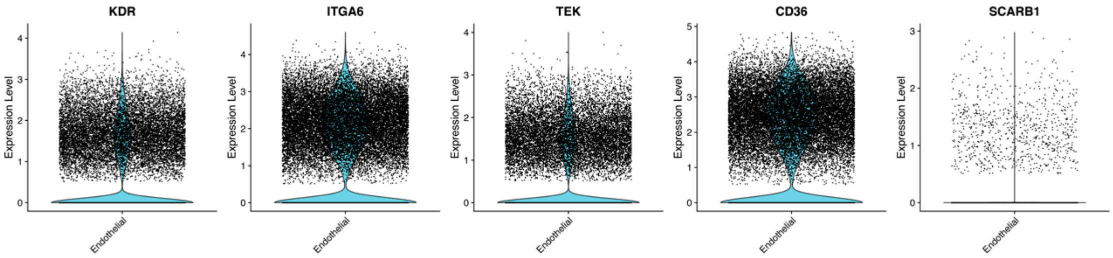

B

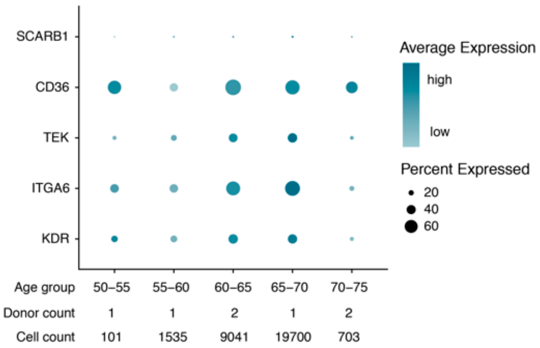

Supplementary Figure. S8 Analysis of single-cell transcriptome data of healthy human hearts.

A Expression of 5 cardiac EC receptor genes in the human hearts (31080 ECs in total).

B Expression of 5 cardiac EC receptor genes from 7 healthy human in different ages.

Supplementary Figure S9

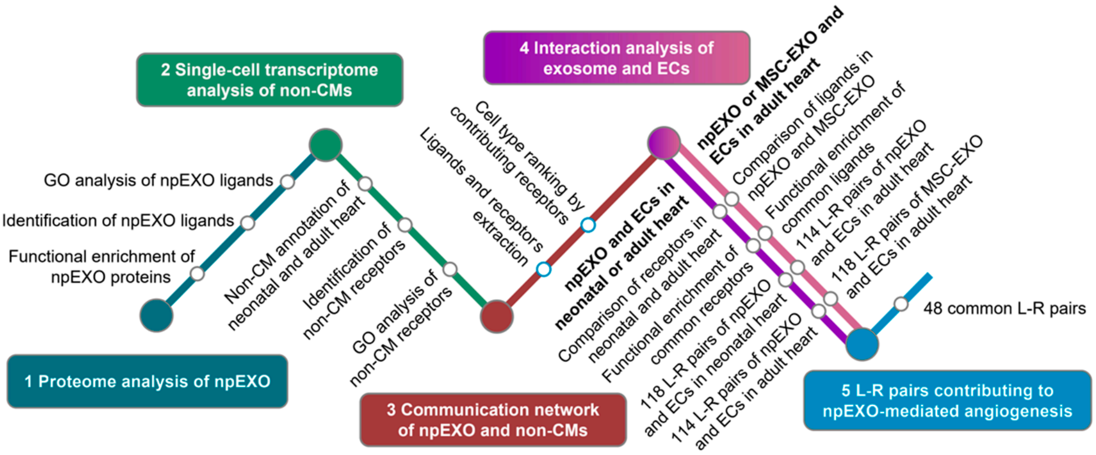

Supplementary Figure. S9 Step-by-step procedures of bioinformatics analysis in this study. The npEXO proteome analysis, including functional enrichment of total proteins or ligands (**Step 1**); single-cell transcriptome analysis of non-CMs and functional enrichment of their receptors respectively in the neonatal and adult mouse heart (**Step 2**); construction of communication network among npEXO ligands and non-CM receptors, including ligands and receptors extraction and the subsequent cell type ranking (**Step 3**); interaction analysis among npEXO ligands and cardiac EC receptors respectively in the neonatal and adult mouse heart, in parallel with interaction analysis among npEXO or MSC-EXO ligands and cardiac EC receptors in the adult mouse heart (**Step 4**); identification of 48 L-R pairs contributing to the npEXO-mediated angiogenesis (**Step 5**).
